# Defining household exposure to the food environment: A comparison of measures based on residential area and activity space

**DOI:** 10.1371/journal.pone.0329442

**Published:** 2025-08-01

**Authors:** Simon Vonthron, Hélène Charreire, Marlène Perignon, Pascaline Rollet, Daisy Recchia, Caroline Mejean, Christophe-Toussaint Soulard, Coline Perrin, Surfood-Foodscapes study group

**Affiliations:** 1 INNOVATION, Univ Montpellier, CIRAD, INRAE, Institut Agro, Montpellier, France; 2 MoISA, Univ Montpellier, CIRAD, CIHEAM-IAMM, INRAE, Institut Agro, IRD, Montpellier, France; Villanova University, UNITED STATES OF AMERICA

## Abstract

The food environment’s impact on food behaviors is widely recognized, yet there is no consensus on how to measure individual exposure, potentially leading to inconsistent results. This study aimed to assess how the relationship between food environment exposure and household characteristics differs based on whether exposure is measured solely via buffers around the home or extended to cover activity spaces. Data from the Mont’Panier cross-sectional study, comprising 699 households in Montpellier city-region, France, were analyzed. We used multivariate multinomial regression models and multiple logistic regression models to explore the associations between household characteristics and five indicators of households’ food environment exposure: number of food stores, number of restaurants, diversity of food stores, relative density of stores selling fruits and vegetables, and relative density of fast-food restaurants. Exposure was measured using two methods: (i) 500m network-buffers around household’s home; and (ii) activity spaces delimited using the daily path area method. Our findings confirm that social disparities in food environment exposure can vary based on the defined exposure area. Variables such as ‘employment status’ and ‘education’, not associated with indicators of food environment exposure around the home, show associations in the activity space. Conversely, ‘age’ and ‘car ownership’ are associated with various indicators of food environment exposure around the home but not in the activity space. Additionally, our analysis reveals that the abundance and diversity of food outlets tend to decrease as household’s distance from the city center increases. Combining measures of food exposure (residential area, activity space) presents a promising approach to understanding food environment dynamics. Future research should investigate whether and how the choice of exposure measures affects food behaviors relative to households’ socioeconomic characteristics. Lastly, we propose two avenues for reflection concerning public policy.

## 1. Introduction

The food environment, encompassing the availability of food outlets within a specific area, is recognized as a significant influencer of food behaviors [1–4]. However, the lack of consensus on how to measure individual exposure poses challenges in comparing studies and may result in inconsistent findings [5–7]. Spatial analysis serves as a prevalent method for evaluating the food environment [8]. Nevertheless, the absence of a standardized approach [9–12] and the variability in spatial units chosen to measure exposure can significantly impact the outcomes of statistical analyses. This phenomenon is referred to as the Modifiable Area Unit Problem (MAUP) [13].

Particularly, the relationship between food behaviors and exposure to the food environment may vary depending on the approach chosen to define the exposure area [14,15]. In addition to place-based approaches that define the food environment of a city, a neighborhood, or a census track, there are people-based approaches used to measure individuals’ real exposure to the food environment around the places they regularly visit [16,17]. However, a significant limitation of these studies is the common use of home-centric measures [18]. These studies typically characterize food exposure based on residential administrative neighborhoods, census spatial units, or buffer zones around individuals’ homes [5,9–11]. Not accounted for are the “areas within which people move or travel in the course of their daily activities” [19], referred to as activity spaces [17,20,21]. Yet, individuals often shop for food outside their residential neighborhoods, particularly during commutes [22,23]. Activity space approaches, therefore, “provide a more accurate assessment of an individual’s daily exposure to food sources and are being recognized as the preferred best-practice within the field” [5]. However, despite the development of mobility-based approaches to precise the space exposure such as those based on GPS, evidence of association between food environment exposure and diet is still inconsistent [24].

There is limited research on the impact of using activity space and home-centric measures to assess food environment exposure, and the relative value of both methods. Li and Kim [25] observed a greater number of stores selling fruits and vegetables in urban areas compared to rural areas when employing buffers around the home. However, they found no difference when using activity space measures. In contrast, Raskind et al. [26] reported no distinction in the association between the food environment and body mass index regardless of whether the food environment was assessed through activity space or home-centric measures. Conversely, Drewnowski et al. [27] and Marwa et al. [28] argue that the choice of measure does matter, even though the last found no correlation between exposure and the use of food outlets. Furthermore, activity space measurement encompasses various methods, including daily path area, minimum convex hull polygons, standard deviational ellipse (SDE), and Kernel density estimation, each with its distinct strengths and limitations [29,30]. For instance, the SDE method employed by Li and Kim [25] seems to be a suitable choice for identifying potentially accessible environments. However, other methods like the daily path area are better suited for measuring the environments to which people are actually exposed [29].

The aim of this paper is to evaluate how definitions of food environment exposure based on households’ characteristics differ when utilizing home-centric measures (buffers around the home) versus activity space approaches. We compared various measures of households’ food exposure using a population sample in the Montpellier city-region, France.

## 2. Methods

### Study population

The Montpellier city-region, situated in the south of France, encompasses 31 municipalities with a combined population of 472,217 inhabitants as of 2017, with approximately 60% residing in the principal city, Montpellier. This region is characterized by relatively high poverty rates, with a poverty rate of 20% for the overall city-region in 2018, and a notably higher rate of 27% within the city of Montpellier itself, according to data from INSEE (the National Institute of Statistics and Economic Studies).

Analyses were performed using data from a cross-sectional study we conducted, known as Mont’Panier. It aimed to explore the relationship between foodscapes and people’s food purchasing behaviors in the Montpellier city-region (https://www.foodscapes.fr/en/project/mont-panier-survey). From May 1, 2018 to December 31, 2019, we recruited 699 households from the city-region. Participants were solicited through a call for participation. Eligible participants had to be 18 years or older, reside in the Montpellier city-region, and be involved in their household’s food shopping. For sampling, we utilized sociodemographic data sourced from the national statistical institute INSEE for the Montpellier city-region. Quota sampling was then performed based on household composition (one adult, multiple adults, one adult with at least one child, and multiple adults with at least one child), as well as the age of the head of the household (< 35 years, 35–50 years, and > 50 years).

The Mont’Panier study was conducted in compliance with the guidelines outlined in the Declaration of Helsinki. All procedures were approved by the Institutional Review Board of the French Institute for Health and Medical Research (IRB Inserm no. IRB00003888 IORG0003254 FWA00005831) and registered with the French *Commission Nationale Informatique et Libertés*. Prior to participation, written electronic informed consent was obtained from each participant after providing a comprehensive explanation of the study. As a token of appreciation, participants received a €15 voucher upon returning all completed data collection materials.

### Definition of food environment exposure areas

The definition of food environment exposure areas involved two measures: (i) buffers around households’ homes and (ii) activity space.

All household data were collected via the Mont’Panier online questionnaire. Exposure areas were delineated based on households’ self-reported major anchor points and transportation modes. In addition to the home, households could identify up to two other anchor points per adult, representing activity locations (such as work, sport facilities, children’s school, etc.) visited at least once a week. For each location, participants specified the most frequent transportation mode used. Notably, anchor points did not include food outlets to mitigate selective daily mobility bias [31,32]. Anchor points were geocoded using the Base Adresse Nationale in February 2020. The Base Adresse Nationale is an official national database listing all addresses in France, and is available in opendata (https://adresse.data.gouv.fr/outils).

### Area around home

While consensus on buffer size for characterizing the food retail environment [5,7] remains elusive, a widely adopted threshold of 500 meters around the home is commonly utilized to delineate the environment potentially influencing people’s behaviors [33]. Consequently, the area of food environment exposure around the home was defined using 500m network-buffers. These network-buffers were computed using the *Hqgis extension (*https://github.com/riccardoklinger/Hqgis*)*.

### Activity space

The household’s activity space was defined by aggregating the activity spaces of adults contributing to the household’s food provisioning. Activity spaces were delineated using the daily path area method. This method consists of delimiting an activity space using buffers defined around anchor points (home, work place, school, etc.) and tracks [29]. This method was chosen for calculating activity space, as previous work has shown that it provides a more accurate estimate of the space used by individuals than other methods such as standard deviational ellipses and, minimum convex hull polygons [34,35], and is particularly used and well-suited to quantifying the accessible outlets both around visited locations and specific routes and thus, it improves the estimate of the influence of the food environment on food behavior [29,36].

Households’ activity spaces encompass the areas surrounding three types of locations: homes, activity sites, and transit routes. These spaces comprise the vicinity of the household’s residence, the surroundings of reported activity locations by each participant within the household, and the regions traversed along the routes from home to these activity sites. In cases where the same adult frequented two activity locations using the same mode of transportation, we incorporated the area along the route connecting these locations. Based on the households’ input, their activity spaces were computed according to 10 scenarios outlined in S1 Appendix. Although a few studies have tested time-weighted activity spaces, particularly regarding air pollutant exposure [37,38], we chose not to rank the activity locations reported by individuals. However, such approaches rely on an assumption we do not share: that the time spent by an individual at a location is associated with frequenting nearby places. In contrast, J. Vallée specifically discusses the importance of weak places [39]. Thus, we assume equal weighting of anchor points.

Regarding the areas surrounding the home, we generated 500 m network-buffers to outline the regions around the anchor points, utilizing the *Hqgis extension*. The routes between anchor points were calculated using the *Openrouteservice API (*https://openrouteservice.org/*)*, employing the fastest path method.

Buffers around routes varied depending on the reported mode of transportation [40].

- For walking and cycling trips, 100m Euclidian buffers were used. This distance is considered appropriate for capturing the environment pedestrians and cyclists encounter during their journeys [40–43].
- For car, motorcycle, and scooter trips, 300m Euclidian buffers were employed. This distance was determined based on our measurement of the Euclidean distance from hypermarkets (notably visible due to their size) to the nearest main road. In our study area, all hypermarkets are located approximately 200 to 300m from the nearest main road.
- Public transit trips were excluded due to unavailability of data. However, the departure and arrival locations were available and use to define the share of activity spaces corresponding to trips made using public transportation. Therefore, public transport is a relatively uncommon mode of transport for the purpose of food shopping [44], including in France where less than 5% of shopping trips (including all types of purchases, not only food-related) are made using public transportation [45], and particularly in context of overcrowding when travelling with groceries [46].

**Fig 1:**
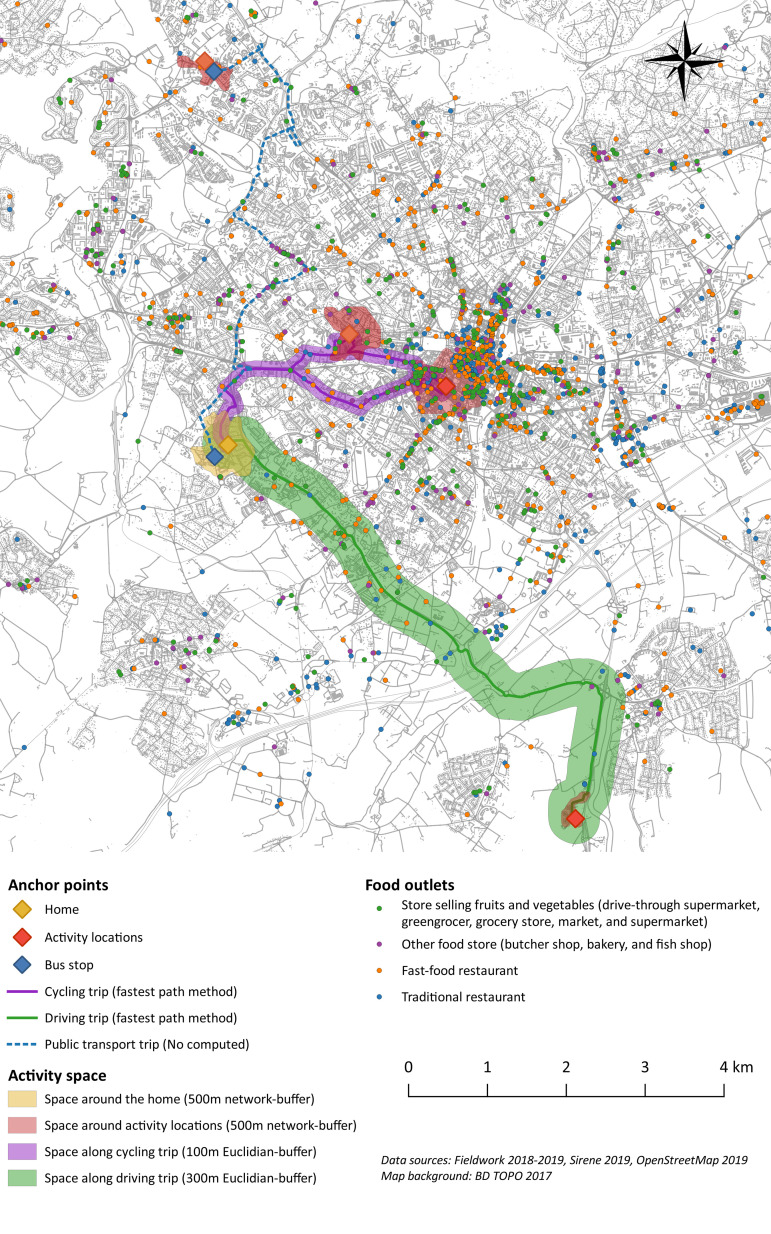
Example of activity space definition.

An example of food exposure area based on activity space is depicted in Fig 1.

The definition of activity space, calculation of its area, and food environment measures were conducted using QGIS 3.4.

The map illustrates an example of the activity space of a household in which two adults participate in grocery shopping. This household resides at the yellow diamond location. One of the two adults typically visits two activity locations (marked in red), using the same mode of transportation: a bicycle (represented by a purple path). The other adult also regularly visits two activity locations. One of these locations, situated to the south, is accessed by car (green path). The other location, to the north, is accessed by bus (dotted blue path). Thus, the household’s activity space consists of a total of seven exposure areas: one yellow zone representing the residential neighborhood, four red zones representing the neighborhoods surrounding the activity locations, and purple and green zones representing exposure areas during the adults’ commutes.

### Database of food outlets and assessment of the food environment

To characterize the food environment, we compiled a database of food outlets utilizing public institutional data and additional sources. INSEE produces the national business register known as ‘Sirene’, which documents and gathers economic and legal information on all newly established businesses, including food outlets. When an individual declares a new business, they propose a code for their principal activity, referred to as APE (Activité Principale Exercée). INSEE then selects the final APE code based on a classification system (NAF Rev.2) defined by decree.

From the database of food outlets encompassing food stores and restaurants, we extracted Sirene data for 14 APE codes (S2 Appendix) dating back to January 2019. Following a reliability assessment of the Sirene data [47], we refined the database through several steps. Duplicate entries were eliminated, and verification was conducted using *Google Street View*. Geocoding of the database was facilitated through *Mon Géocodeur 2.5,* with further refinement achieved using *Google Maps*. Additionally, markets, which were not covered by Sirene, were sourced from OpenStreetMap and integrated into the database. Extensive online searches were conducted on platforms such as Google Maps, corporate websites of major food retailers, and municipality websites (providing information on local markets) to bolster the database’s reliability. Final validations were conducted through field observations covering approximately 7% of the study area. Food stores were categorized into 8 distinct types, including butcher shops, bakeries, drive-through supermarkets, fish shops, greengrocers, grocery stores, markets, and supermarkets. The three restaurant categories available in the database were grouped into two categories (S2 Appendix). Cafeterias are a type of fast food restaurants commonly found in France in shopping centers and highway rest areas. They were therefore included in the fast-food restaurants category, allowing for the calculation of relative fast-food densities (see the following paragraph).

The assessment of food environment exposure involved the utilization of 5 indicators for each exposure area (buffer around home and activity space): number of food stores, diversity of food outlets (ranging from 0 to 8 food store categories), relative density of stores selling fruits and vegetables (ratio of fruit and vegetable (F&V) retailers, including drive-through supermarkets, greengrocers, grocery stores, markets, and supermarkets, to total food stores), number of restaurants, and relative density of fast food restaurants (ratio of fast food restaurants to total restaurants). To incorporate these indicators into our regression models, tertiles were used for the numbers of food stores and restaurants. Three diversity ranges were identified based on distribution (0–2; 3–5; 6–8). The cutoff point for relative densities was set at 50% (<and>=). In cases where the ratio calculation involved missing values due to the absence of food stores or restaurants, these missing values were considered as ‘less than 50%’.

### Household characteristics

Household characteristics were collected using a questionnaire.

The household characteristics considered in the analysis were as follows: age of household head (<35 years; 35–50 years; > 50 years), education level of household head (high school diploma or lower; undergraduate degree; postgraduate degree), household composition (one adult; multiple adults; one adult with at least one child; multiple adults with at least one child), employment status of household head (divided into employed (including ‘employee’ and ‘self-employed’) and unemployed (including ‘jobless’, ‘homemaker’, ‘retired’, and ‘sick leave’), as well as students), household income per consumption unit (categorized into quartiles based on the income distribution of the Montpellier city-region population: <980, 980-1722, 1723-2550 and >2550€/month), car ownership (yes or no), and home location (Montpellier city-center; pericentral neighborhoods of Montpellier city, and peri-urban areas). The “home location” variable was not directly collected via a questionnaire. Instead, the participants’ addresses were collected. The variable was filled in from this address using QGIS 3.4.

### Statistical analysis

Descriptive statistics were presented as means (standard deviation) for continuous variables and percentages for categorical variables. Multivariate regression models were employed to examine the associations between households’ characteristics and the size of area of exposure (multiple linear regression models), and their food environment exposure (multivariate multinomial regression models and multiple logistic regression models) in each exposure area (buffer around home and activity space). Multivariate multinomial regression models were utilized to calculate odds ratios (ORs) and corresponding 95% confidence intervals (CIs), assessing the strength of associations between the numbers of food stores, restaurants, or diversity of food stores, and household characteristics. Multiple logistic regression models were also employed for the relative densities of fast-food restaurants and F&V stores. Covariates (households’ characteristics) associated with food environment exposure indicators at a significance level of 0.2 in bivariate analyses were included in subsequent multivariate models. The risk of multicollinearity was addressed by examining the generalized variance inflation factors (GVIF) of each model, with an adjusted GVIF below five considered acceptable. Normality of residuals has been verified for multiple linear regression models using qqplots.

All analyses were conducted on the weighted sample in order to ensure the representativeness of the sample in terms of socioeconomic diversity. Weights were calculated using the raking ratio method to ensure that the marginal distribution of the weighted sample aligns with that of the targeted population. Sociodemographic data for the Montpellier city-region were sourced from the French census database (INSEE) of 2017. The sample was adjusted through calibration on margins based on income per unit of consumption and household composition, cross-referenced against the household head’s age group. Statistical analyses were performed using *R 4.2.1,* with the threshold for statistical significance set at p < 0.05.

The results of all statistical analyses are presented in graphical form in the subsequent section. Additional details can be found in S3 Appendix.

## 3. Results

### Sample characteristics

The characteristics of the analytic sample are outlined in Table 1.

**Table 1:** Descriptive statistics for the analytic sample (n = 699).

Household characteristics	Raw sample		Weighted sample
	n	%	%
**Age of household head**			
“ < 35 years	240	34	29
35 to 50 years	212	30	26
> 50 years	247	35	45
**Education level of household head**			
High school diploma or lower	171	25	24
Undergraduate degree	258	37	35
Postgraduate degree	270	39	41
**Household composition**			
One adult	232	33	43
Multiple adults with at least one child	168	24	21
Multiple adults	264	38	26
One adult with at least one child	35	5	10
**Employment status of household head**			
Employed^1^	493	71	69
Unemployed^2^	133	19	22
Student	73	11	9
**Household income per consumption unit**			
< 980 €/month	210	30	24
980 to 1,722€/month	179	26	24
1,723–2,550 €/month	138	20	21
> 2,550 €/month	123	18	24
Refuse to answer	49	7	7
**Home location**			
Montpellier city-center	130	19	18
Pericentral neighborhoods of Montpellier	287	41	41
Peri-urban area	282	40	41
**Car ownership**			
No	133	19	19
yes	566	81	81

^1^ *Listed as ‘employee’, and ‘self-employed’*

^2^ *Listed as ‘jobless’, ‘homemaker’, ‘retired’, and ‘sick leave’*

### Size of area of exposure

**Fig 2:**
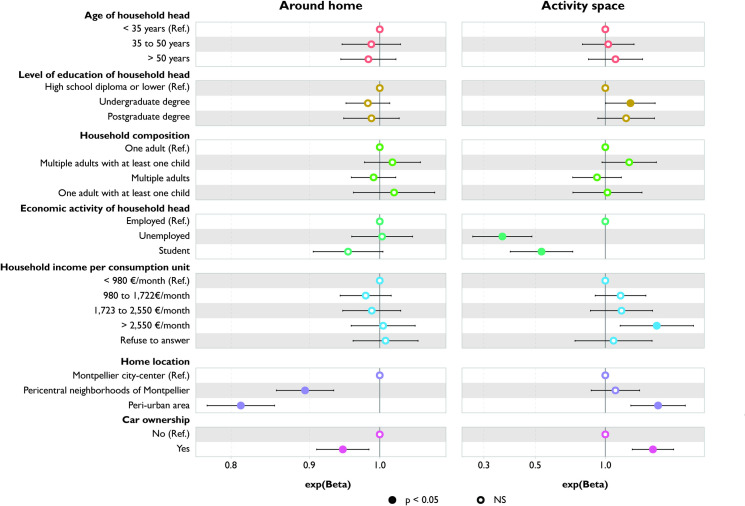
Variation in area of exposure according to household characteristics: comparison between around home and activity space measures.

Compared to other households, the area of exposure around the home is significantly smaller for households residing in pericentral neighborhoods or peri-urban municipalities and for those who own cars (Fig 2). Conversely, the activity space is larger for households living in peri-urban municipalities and for car owners.

There is no significant difference in the size of the area of exposure around the home based on household characteristics such as education level, income level, and employment status. However, when considering the entire activity space, households whose head holds an undergraduate degree and the wealthiest households have a larger area of exposure. In contrast, students and households with unemployed heads have smaller areas of exposure.

### Number of food stores

**Fig 3:**
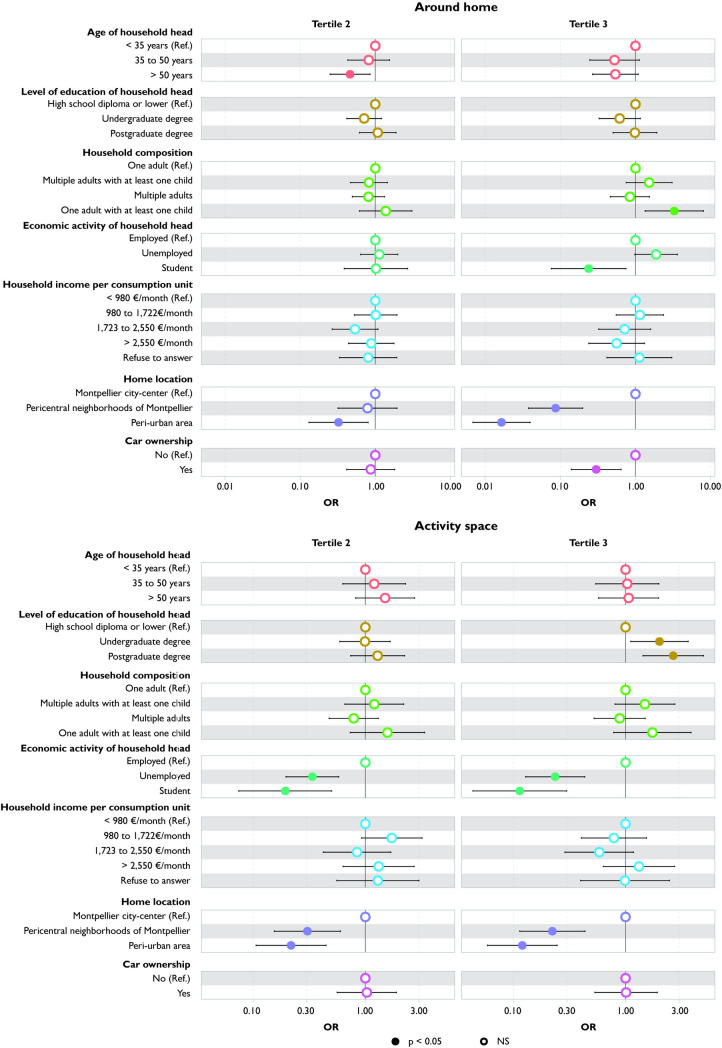
Variation in number of food stores according to household characteristics: comparison between around home and activity space measures.

Students are exposed to a significantly lower number of food stores, both around their home and in their activity space, compared to households with employed heads, with the disparity being even greater in the activity space (Fig 3). There is no difference in exposure around the home between households with employed and unemployed heads. However, when considering activity space, households with an unemployed head are exposed to a significantly smaller number of food stores compared to those with employed heads.

There is no difference in exposure around the home based on education level. However, when considering the entire activity space, households whose heads hold a postgraduate degree are exposed to a higher number of food stores compared to those with the lowest education level (high school diploma or lower).

Households residing in pericentral neighborhoods and peri-urban areas are exposed to a smaller number of food stores around their home compared to those living in the Montpellier city-center. These associations remain significant when considering activity space.

Only when considering the highest tertile of the number of food stores, car owners are exposed to fewer food stores around their homes than households without a car; and compared to single adults, single-parent families are more exposed to a greater number of food stores around their home. However, this difference is no longer significant when assessed across the entire activity space.

### Number of restaurants

**Fig 4:**
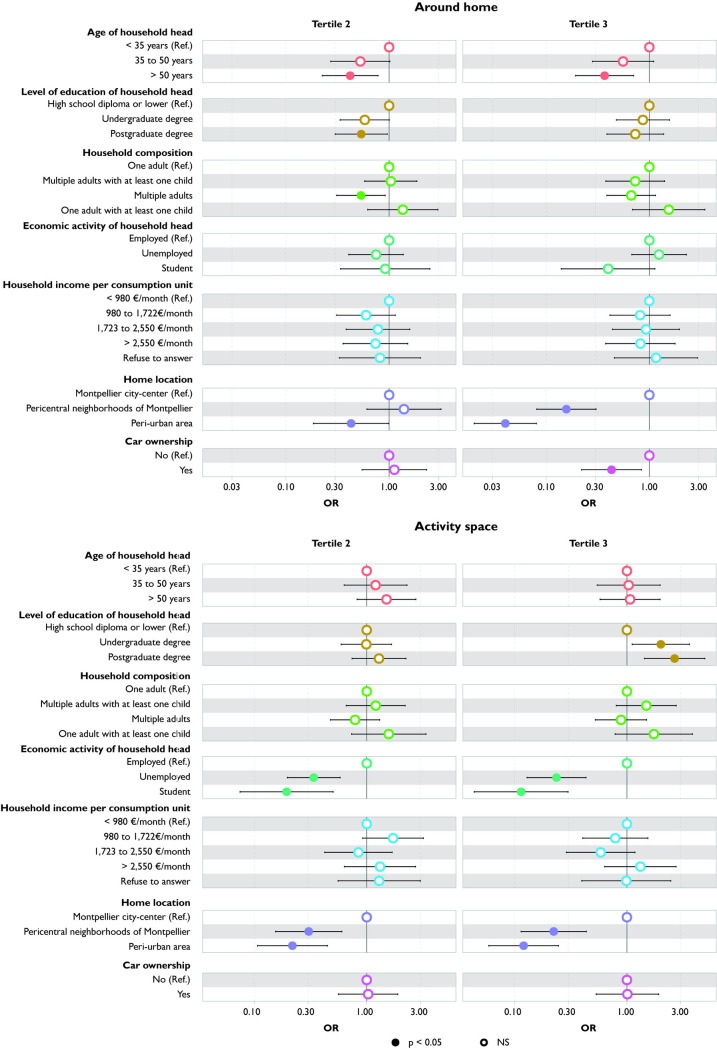
Variation in number of restaurants according to household characteristics: comparison between around home and activity space measures.

Households with a household head aged over 50 are exposed to fewer restaurants around their home compared to those with younger heads (<35 years) (Fig 4). Car owners also encounter fewer restaurants around their home. However, these differences cease to be significant when the activity space measure is employed.

Conversely, although no significant differences are observed between household with student or unemployed head and household with employed head in terms of number of restaurants around their home, both household with student and unemployed head are exposed to fewer restaurants within their activity space.

Households residing in pericentral neighborhoods and peri-urban areas are exposed to a smaller number of restaurants compared to those living in the Montpellier city-center. These associations persist when considering activity space.

### Diversity of food stores

**Fig 5:**
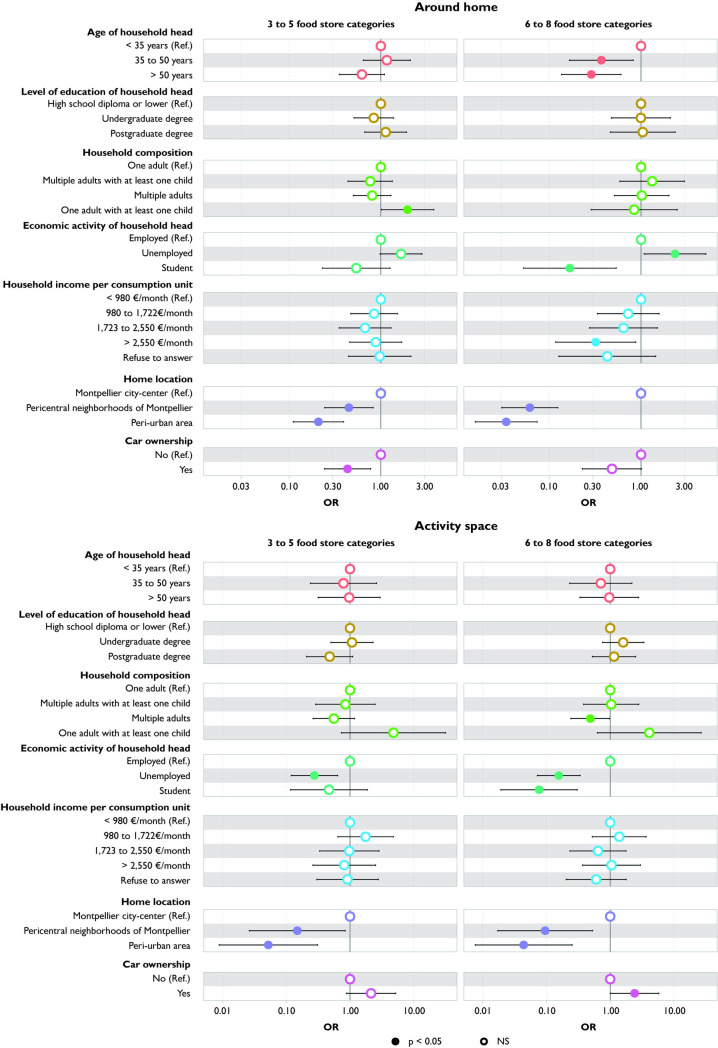
Variation in diversity of food stores according to household characteristics: comparison between around home and activity space measures.

Households with a household head aged over 35 and car owners are exposed to a narrower range of food stores around their home compared to those with a younger household head (<35 years). However, these differences cease to be significant when their entire activity space is taken into account (Fig 5).

Households with an unemployed head are exposed to a slightly higher diversity of food stores around their home compared to households with an employed head. This relationship reverses when considering their activity space. Students are exposed to a narrower range of food stores compared to employed households, both around their home and in their entire activity space.

Households residing in pericentral neighborhoods and peri-urban areas are exposed to a narrower range of food stores compared to those living in the Montpellier city-center. These associations persist but become weaker when considering activity space.

### Relative density of stores selling fruits and vegetables

**Fig 6:**
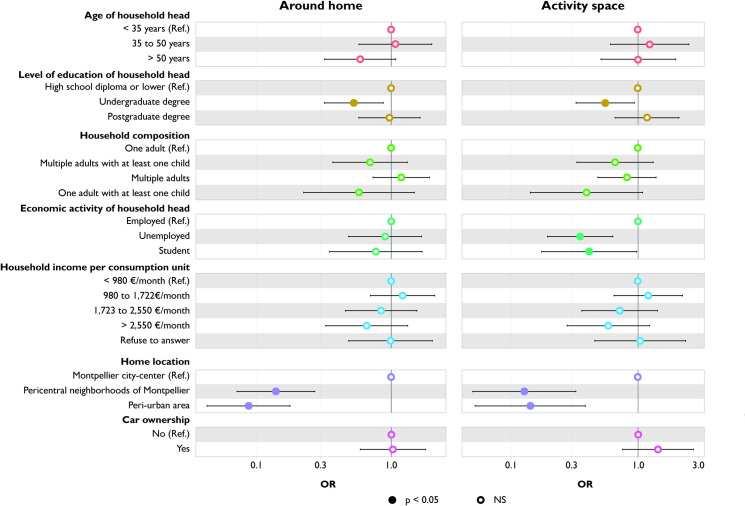
Variation in relative density of F&V stores according to household characteristics: comparison between around home and activity space measures.

Households whose heads have undergraduate degree are exposed to a lower relative density of stores selling fruits and vegetables, both around their home and in their activity space, compared to households with the least educated heads (Fig 6). Results for households living in pericentral neighborhoods and peri-urban areas are similar to those of households living in the Montpellier city-center.

There is no significant difference in exposure around the home based on the employment status of the household head. However, considering the entire activity space reveals significantly lower exposure for households with student and unemployed heads compared to those with employed heads.

### Relative density of fast-food restaurants

**Fig 7:**
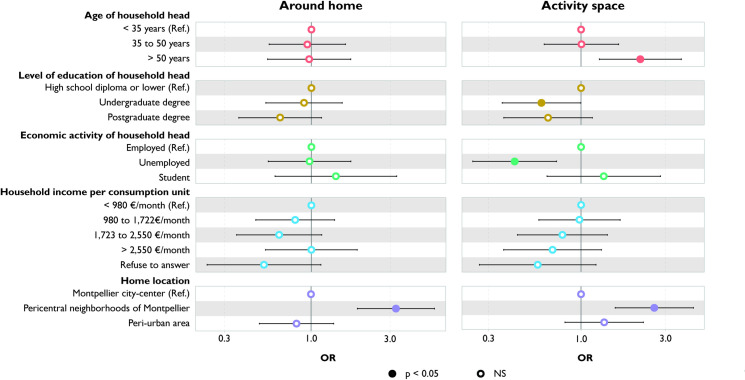
Variation in relative density of fast-food restaurants according to household characteristics: comparison between around home and activity space measures.

Households with a household head over 50 are exposed to a higher relative density of fast-food restaurants in their activity space than those whose household head is younger (<35 y) (Fig 7). Conversely, households whose heads have an undergraduate degree or are unemployed are exposed to a lower relative density. However, around the home, there is no significant difference in exposure according to age, education level, and employment status of the household head.

Households living in pericentral neighborhoods are exposed to a higher relative density of fast-food restaurants than those living in the Montpellier city-center or in peri-urban areas, both around their home and in their activity space.

## 4. Discussion

### Effect of exposure area definition on food environment exposure

Our findings confirm that social disparities identified in food environment exposure may vary depending on the method used to define the exposure area. Characteristics such as ‘employment status’ and ‘education level’ (highest) that are associated with almost any indicators of food environment exposure around the home appear to be associated with all indicators of food environment exposure in the activity space. Conversely, ‘age’ (oldest) and ‘car ownership’ are associated with various indicators of food environment exposure around the home but not in the activity space. These results help elucidate the inconsistencies found in food environment studies [5–7]. By highlighting the impact of exposure area definition on food environment exposure, our findings contribute to the literature on the MAUP. Specifically, by showing the effect of adopting an activity space approach rather than a home-centric one, our results complement those of the recent literature review of Chen et al. [14], which did not encompass activity space approaches.

Furthermore, our findings resonate with the Neighborhood Effect Averaging Problem (NEAP) described by Kwan [48], indicating that overlooking daily mobility can lead to inaccurate exposure assessments. For instance, social disparities in exposure to air pollution may go unnoticed when daily mobility is disregarded, especially concerning employment status [49]. Likewise, the relationship between individuals’ health behaviors and the medical environment around their home may differ based on their daily mobility patterns [50]. Thus, our results align with previous food environment research employing alternative exposure area definition methods. Kestens et al. [51] showed that activity space-based measures of food environment exposure were significantly and more strongly associated with overweight than home-centric measures of exposure. Additionally, Li and Kim [25] observed that associations between food environment exposure and food shopping patterns varied significantly based on population density in residential neighborhoods but not in the activity space.

Nonetheless, home centric measures are often used as proxy because activity space data are rarely available and more difficult to collect [5]. Our results call into question the relevance of this widespread use. However, it is possible that this practice is justified for specific social groups (unemployed individuals, students, city center residents, and carless households) or types of measures (relative densities), as suggested by the relatively high correlations observed between measures of food environment exposure around the home and within the activity space (Table 7 in S3 Appendix). Households living in Montpellier and those without a car also tend to have a relatively higher exposure area compared to others. This may be explained by the fact that these households reside in areas with a denser road network (Fig 2) and a closer proximity between their homes and activity locations. Regarding unemployed households and students, the very low correlation between the exposure area sizes suggests that, although they travel less than employed individuals (Fig 2), they remain mobile. This indicates that their activity locations and the routes they take are less equipped with food outlets. This is particularly understandable for students, whose living spaces are concentrated in relatively homogeneous areas, such as student neighborhoods with dedicated residences and university campuses that lack commercial facilities—these being their main activity locations. It is thus possible that, for many students and unemployed households, more than for other social groups, the food environment they are exposed to is primarily limited to that around their residence. However, caution is required regarding students, as this observation may also result from a methodological artifact—a lack of consideration for establishments located near universities for students using public transport (which is not accounted for in this study). Moreover, these correlations are even stronger when using relative density measures, for which the correlations between the measures around the home and within the activity space are generally higher. Thus, the use of home-based buffers appears to be a better proxy for relative density measures and for measures concerning households residing in dense urban areas, carless households, unemployed households, and, potentially, students.

In addition, utilizing exposure around the home remains a valuable approach for uncovering associations based on proximity. For instance, our findings demonstrate that car owners are exposed to fewer food outlets and less diverse food stores around their homes. This result is particularly noteworthy as it raises critical questions regarding public policies, such as those related to public transit. Barriers to accessing healthy food may be linked to socioeconomic characteristics [52]. Therefore, while a poor food environment around the home may not pose a problem for individuals with access to a car, enabling them to travel outside their neighborhood, it could become a concern when individuals are no longer able to drive due to health, age, or economic reasons. Hence, the food environment around the home still warrants specific research attention.

Therefore, considering that associations between food environment exposure and households’ characteristics may vary depending on the method used to delineate of space for measuring food exposure, we recommend that future research on food environment exposure utilize both activity space and home-centric measures whenever feasible.

### Associations between food environment exposure and household characteristics

Our findings regarding the associations between food environment exposure and households’ characteristics indicate that the abundance and diversity of food outlets to which households are exposed generally diminish as the distance from the city center increases. These results remain consistent even when considering activity space, despite households residing in peri-urban areas having larger activity spaces. This contrasts with the findings of Li and Kim [25] in Cincinnati, Ohio, USA, who observed a greater number of F&V stores in urban areas compared to rural areas around the home, but found no difference when using the activity space approach. The divergence in our results could be attributed to disparities between US and French cities in terms of urban morphology and the spatial organization of retail activities. Specifically, we hypothesize that the hyper-centralization of retail food supply in the Montpellier city-center [53] could account for the differences in exposure based on home location: the daily mobility of households may not adequately compensate for this spatial concentration of retail food supply.

Our second notable finding is that households’ sociodemographic characteristics exhibit limited associations with food environment exposure, except for employment status and car ownership. Specifically, being a student or unemployed is linked to a less abundant and diversified food environment both around the home and within the activity space. Additionally, students are exposed to relatively fewer stores selling fruits and vegetables within their activity space. Similarly, households with an unemployed head are exposed to relatively fewer fast-food restaurants in their activity space. These disparities in absolute exposure (measured by the number and diversity of food outlets) within the activity space associated with employment status may be attributed to the smaller areas of exposure for students and unemployed households. Moreover, it suggests that the areas frequented by these households during their daily activities have fewer food outlets. For instance, within French university campuses, there are typically no food stores available.

Car ownership is associated with a smaller number and diversity of food outlets around the home. This aligns with the observation that households without a car often reside in neighborhoods with a higher density of shops [53]. Additionally, car owners tend to have larger activity spaces compared to non-car owners. However, when considering the entire activity space, the food environment exposure of car owners does not significantly differ from that of households without a car, except for a higher exposure to fast-food restaurants among the total number of restaurants in their activity space. This result can be explained by the fact that households with a car tend to live in residential areas where there are few stores but many parking opportunities (e.g., street parking, private driveways). However, for these car-owning households, the food environment of the areas they reside in may reinforce their dependence on the car for grocery shopping. Nevertheless, car ownership enhances their accessibility, as greater reliance on their car reduces their dependence on the local food environment. The issue arises, however, for households that own a car but face constraints in using it, particularly due to financial limitations or driving ability (see above).

This finding contrasts with the results of Burgoine and Monsivais [40], who used a similar delineation method for activity space in Cambridgeshire, UK, and found that households without a car were exposed to fewer food outlets and to less diverse food stores in their activity space. However, their analysis, unlike ours, was not adjusted for the home location of participants within the city. Thus, we hypothesize that these differences may be attributed to variations in the spatial distribution of food outlets between Cambridgeshire and Montpellier. Consequently, it would be advisable to supplement people-based studies with place-based studies that emphasize the influence of urban structure and neighborhood characteristics on individuals’ food environment [54].

### Strengths and limitations

One of the primary strengths of our study lies in the comprehensive characterization of the food environment through the utilization of both absolute and relative measures. While many studies typically focus on a single type of outlet, such as fast-food restaurants or supermarkets [55], and predominantly employ absolute measures like counts or presence, our approach incorporates both absolute and relative measures. Although relative measures are less commonly utilized, scholars such as Clary et al. [56] have shown their significance in food environment research. Nonetheless, Thornston et al. [57] caution that the limitations associated with relative measures may be overlooked. By combining absolute and relative measures, we ensure a more comprehensive understanding of the food environment landscape, allowing for a more nuanced analysis.

Additionally, our study benefits from defining the activity space at the household level, which represents another notable strength. Unlike previous studies that often define activity space at the individual level, our approach at the household level is more appropriate for assessing food environment exposure because many food-related activities are shared among household members [58]. Moreover, our inclusion of a variety of anchor points to delineate household activity spaces, beyond just workplaces, allows for a more comprehensive understanding of individuals’ daily mobility patterns. This approach also considers diverse transportation modes and the routes taken, providing a more accurate depiction of food environment exposure within the household context.

Our study has several limitations, that should be acknowledged. Firstly, as previously mentioned, the lack of detailed data on public transportation routes in Montpellier limited the part of the food environment exposure associated with this mode of travel. This situation applies to, 23% (n = 160) of the households in our sample, at least in part (i.e., at least one of the different activity locations reported by household members taking part in the errands) but only 9% do not own a car. Secondly, the threshold values used to define exposure areas, particularly the 500-meter radius around anchor points may not accurately reflect individuals’ perceptions of their neighborhood size. Variations in neighborhood size perception can be influenced by factors such as socio-economic status, age, attachment to the neighborhood, proximity to amenities, and the built environment [59–63]. Therefore, using fixed delineations like the 500-meter radius may lead to inaccuracies in exposure measurement [33]. Furthermore, our use of a network-buffer approach for defining exposure areas may have introduced variations due to differences in street densities and intersections, potentially affecting the measures of food environment exposure. Additionally, when delineating exposure areas around large places like universities, using buffer zones around centroids may not adequately capture the full extent of surrounding food outlets.

While routes between anchor points were determined using the fastest-path method, it is important to note that individuals may choose to take detours based on various factors such as their mode of transportation [64,65] and the availability of amenities [66,67] along the way. This variability in route selection can result in differences between the environmental characteristics (such as the presence of food outlets) of observed path and computed paths, particularly depending on the mode of transportation used [68]. Additionally, participants in our survey were able to report up to two activity locations for each adult household member. All reported anchor points were given equal weight in determining activity spaces, irrespective of the time spent at each location. This approach contrasts with studies like that of Jankowska et al. [69], which calculated time-weighted spatial exposure measures using Global Positioning System mobility data. They emphasized the challenges associated with using and interpreting such time-weighted measures of exposure. Conversely, Vallée [39] highlighted the importance of occasionally visited places, some of which may play a crucial role in providing individuals with access to resources, including food stores, that may not be available in the areas they frequent more regularly.

The food outlet database we used is based on the Sirene business directory. The main limitation of this database [47] is the persistence of closed outlets, and we made numerous corrections to enhance its reliability. In addition, we used several other local data sources to ensure its comprehensiveness and to incorporate food market that are not available in Sirene. However, the resulting database is not flawless. Specifically, two main limitations persist: food sales as a secondary activity and the categorization of restaurants. (i) The categories do not allow us to characterize restaurants in fine detail and therefore to distinguish fast foods precisely. Nevertheless, in order not to limit ourselves to the main fast-food chains and to include those that do not belong to these chains, we have considered the entire category of the nomenclature. This choice introduces a classification limit, but allows us to better characterize the food environment.

Finally, it is important to exercise caution when generalizing these results to the entire French population, as our study was confined to an urban area in the South of France.

## 5. Conclusion: Future research directions and implications for public policy

Echoing the findings of Sherman et al. [70], who proposed that triangulation of methods could offer a more comprehensive and nuanced understanding of accessibility to healthcare opportunities, integrating measures of food exposure from both residential areas and activity spaces emerges as a promising avenue for addressing food environment challenges.

Despite the ongoing debate regarding the optimal method for measuring people’s food environment, our study highlights how the influence of households’ characteristics on definitions of food environment exposure varies depending on the measurement approach: buffers around home or a broader activity space. Activity space measures offer additional insights that complement those obtained from around home measures, particularly in assessing the association of food environment exposure indicators with characteristics such as car ownership, employment status, or home location. Future research should delve deeper into whether, and under what circumstances, the choice of measures for determining food environment exposure impacts food behaviors as defined by households’ socioeconomic characteristics. Moreover, considering the possible impact of urban structure and the spatial concentration of food outlets in the city center, it would be premature to disregard place-based approaches to studying the food environment in future research endeavors.

In terms of public policy implications, our findings suggest two avenues for consideration. Firstly, a more nuanced understanding of food environment exposure can aid public authorities in pinpointing neighborhoods requiring priority intervention. By identifying areas with disparities in food environment exposure, public authorities can implement strategies to promote the establishment of healthy food outlets in underserved residential neighborhoods [71–74]. These interventions may encompass a range of policies beyond urban planning, including economic incentives and community development initiatives. For example, in the context of urban renewal projects or the construction of new neighborhoods, public authorities can acquire commercial spaces and lease them to selected retailers with the explicit goal of both accommodating the diverse food procurement practices of residents [75] and facilitating the adoption of healthy food behaviors. Secondly, public interventions should be tailored to account for resident’s daily mobility patterns. Drawing parallels with research on depression [50,76], our study highlights that individuals with limited mobility, particularly those residing in disadvantaged neighborhoods [77,78], may experience modified food environment exposure through interventions targeting the residential environment. Although there is little research on their effects, with the exception of Widener’s work in Toronto [79], public transportation policies could help reduce inequalities in food access by decreasing dependence on private cars. This applies both to carless households —who rely on local food retails, public transport, and mutual aid—and to those who own a car but face financial, health, or age-related constraints on its use. However, for people with higher mobility, particularly commuters, interventions solely focused on residential areas may prove inadequate or inappropriate. This underscores the necessity of considering activity spaces in addition to residential environments when designing targeted interventions to address food environment disparities.

## Supporting information

S1 Appendix Situations used to compute activity spaces. (DOCX)

S2 Appendix APE codes extracted and reclassification of food outlet categories. (DOCX)

S3 Appendix Statistical results. (DOCX)
