# Supplementary material for: Defining household exposure to the food environment: A comparison of measures based on residential area and activity space
Source: PLoS One. 2025 Aug 1;20(8):e0329442. doi: 10.1371/journal.pone.0329442 (PMC12316281; doi:10.1371/journal.pone.0329442)
Supplement: S1 Appendix — (DOCX) [file pone.0329442.s001.docx]

## S1 Appendix: Situations used to compute activity spaces

| **Situation** | **Composition of the activity space** | **Schematic representation of the activity space** |
| --- | --- | --- |
| (1) | Space around home | 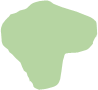 |
| (2) | Spaces around home and around one activity location, as well as those along the route linking the two locations. | 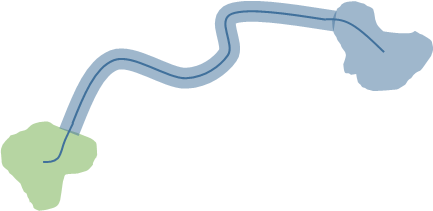 |
| (3) | Spaces around home and around two activity locations, as well as those along the routes of one adult using the same mode of transportation to travel to both locations. | 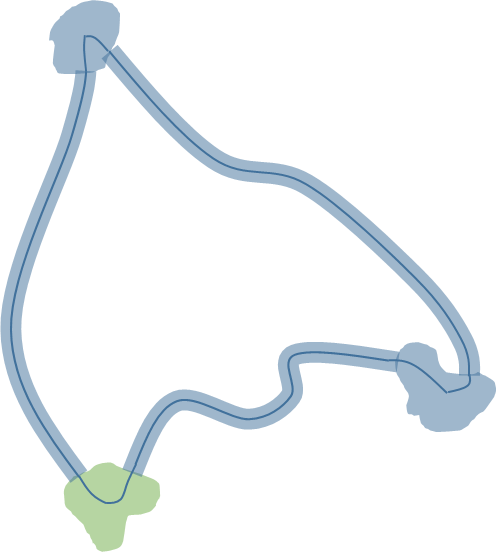 |
| (4) | Spaces around home and around two activity locations, as well as those along the routes of one adult using a different mode of transportation to travel to each activity location. | 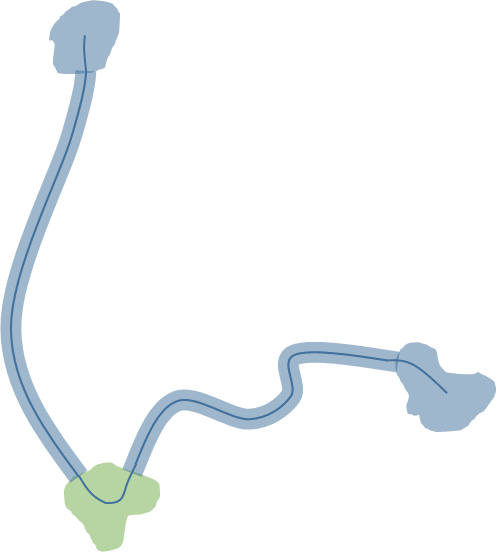 |
| (5) | Spaces around home and around two activity locations of two adults, as well as those along the routes to these locations. | 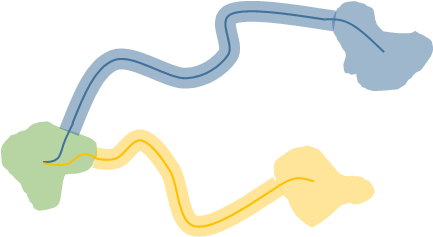 |
| (6) | Spaces around home and around several activity locations, as well as those along the routes to them. At least one adult travels to two activity locations using two different modes of transportation, and one adult travels to one activity location. | 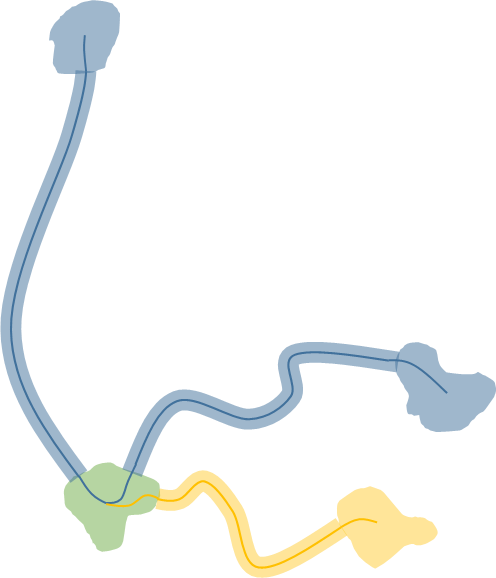 |
| (7) | Spaces around home and around several activity locations, as well as those along the routes to them. At least one adult travels to two activity locations using the same mode of transportation, and one adult travels to one activity location. | 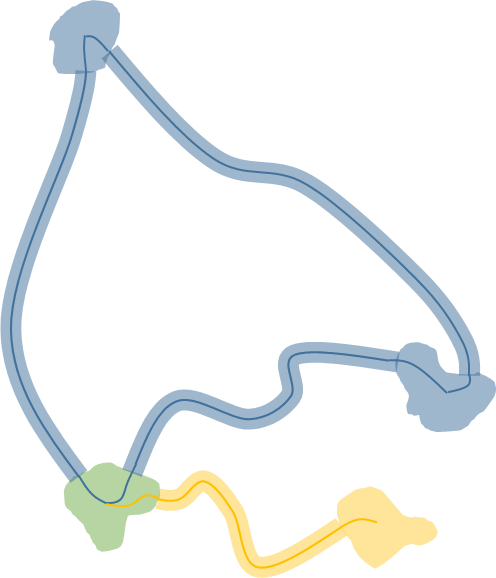 |
| (8) | Spaces around home and around two activity locations per adult (two adults minimum), as well as those along the routes. At least one adult travels to two activity locations using the same mode of transportation, and at least one adult travels to two activity locations using two different modes of transportation. | 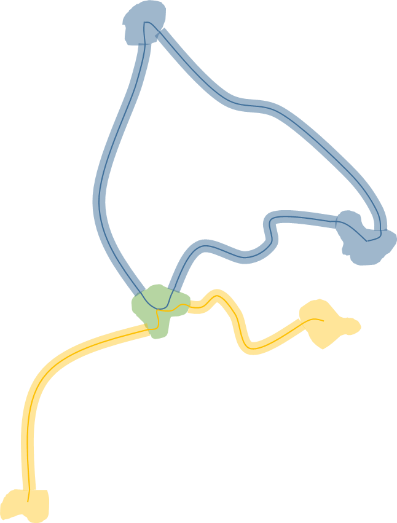 |
| (9) | Spaces around home and around two activity locations per adult (two adults minimum), as well as those along the routes. Each adult travels to two activity locations using the same mode of transportation. | 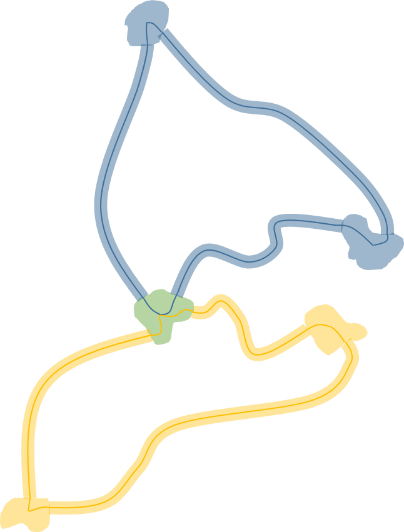 |
| (10) | Spaces around home and around two activity locations per adult (two adults minimum), as well as those along the routes. Each adult travels to two activity locations using two different modes of transportation. | 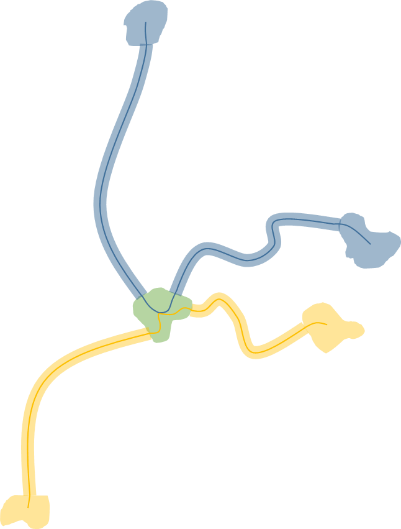 |
