# Supplementary material for: Defining household exposure to the food environment: A comparison of measures based on residential area and activity space
Source: PLoS One. 2025 Aug 1;20(8):e0329442. doi: 10.1371/journal.pone.0329442 (PMC12316281; doi:10.1371/journal.pone.0329442)
Supplement: S2 Appendix — (DOCX) [file pone.0329442.s002.docx]

## S2 Appendix: APE codes extracted and reclassification of food outlet categories

|  | **Sirene** (APE code) | **Standardized category** |
| --- | --- | --- |
| **Food stores** | Butcher shop (47.22Z) | Butcher shop |
|  | Baked goods shop (10.71B)  Bakery (10.71C)  Patisserie (10.71D) | Bakeries |
|  | General food store (47.11B)  Grocery store (47.11C) | Grocery store |
|  | Supermarket (47.11D; 47/11E)  Large supermarket (47.11F) | Supermarket |
|  | Fish shop (47.23Z) | Fish shop |
|  | Greengrocer (47.21Z) | Greengrocer |
| **Restaurants** | Cafeterias and buffets (56.10B)  Fast food restaurants (56.10C) | Fast food restaurant |
|  | Traditional restaurants (56.10A) | Traditional restaurant |
