# Supplementary material for: Defining household exposure to the food environment: A comparison of measures based on residential area and activity space
Source: PLoS One. 2025 Aug 1;20(8):e0329442. doi: 10.1371/journal.pone.0329442 (PMC12316281; doi:10.1371/journal.pone.0329442)
Supplement: S3 Appendix — (DOCX) [file pone.0329442.s003.docx]

## S3 Appendix: Statistical results

Table 1: Characteristics of the raw sample and the study area population according to criteria used for quotas and weighting

|  | **Household characteristics** | **Raw sample %** | **Population of the study area^1^** |
| --- | --- | --- | --- |
| **Criteria used for quotas and weighting** | **Age of household head** |  |  |
|  | < 35 years | 34.3 | 28.7 |
|  | 35 to 50 years | 30.3 | 26.5 |
|  | > 50 years | 35.4 | 44.9 |
|  | **Household composition** |  |  |
|  | One adult | 33.2 | 43.6 |
|  | Multiple adults with at least one child | 24.0 | 20.6 |
|  | Multiple adults | 37.8 | 25.9 |
|  | One adult with at least one child | 5.0 | 9.9 |
| **Criteria used for weighting** | **Household income per consumption unit** |  |  |
|  | < 980 €/month | 30.0 | 25.0 |
|  | 980 to 1,722€/month | 25.6 | 25.0 |
|  | 1,723 to 2,550 €/month | 19.8 | 25.0 |
|  | > 2,550 €/month | 17.6 | 25.0 |
|  | Refuse to answer | 7.0 | / |

^1^ Sociodemographic data for the Montpellier city-region were sourced from the French census database (INSEE) of 2017.

Table 2: Variation in area of exposure according to households’ characteristics: comparison between around home and activity space measures

|  | **Around home** | | | **Activity space** | | |
| --- | --- | --- | --- | --- | --- | --- |
| **Age of household head** | exp(Beta) | 95% CI^1^ | p-value | exp(Beta) | 95% CI^1^ | p-value |
| < 35 years (Ref.) | — | — |  | — | — |  |
| 35 to 50 years | 0.99 | 0.95, 1.03 | 0.6 | 1.03 | 0.80, 1.33 | 0.8 |
| > 50 years | 0.98 | 0.94, 1.02 | 0.4 | 1.11 | 0.85, 1.45 | 0.5 |
| **Education level of household head** |  |  |  |  |  |  |
| High school diploma or lower (Ref.) | — | — |  | — | — |  |
| Undergraduate degree | 0.98 | 0.95, 1.02 | 0.3 | 1.28 | 1.00, 1.64 | 0.046 |
| Postgraduate degree | 0.99 | 0.95, 1.03 | 0.6 | 1.23 | 0.93, 1.63 | 0.15 |
| **Household composition** |  |  |  |  |  |  |
| One adult (Ref.) | — | — |  | — | — |  |
| Multiple adults with at least one child | 1.02 | 0.98, 1.06 | 0.4 | 1.27 | 0.97, 1.66 | 0.084 |
| Multiple adults | 0.99 | 0.96, 1.02 | 0.6 | 0.92 | 0.72, 1.17 | 0.5 |
| One adult with at least one child | 1.02 | 0.96, 1.09 | 0.5 | 1.02 | 0.73, 1.44 | >0.9 |
| **Employment status of household head** |  |  |  |  |  |  |
| Employed^2^ (Ref.) | — | — |  | — | — |  |
| Unemployed^3^ | 1.00 | 0.96, 1.05 | 0.9 | 0.36 | 0.27, 0.48 | <0.001 |
| Student | 0.95 | 0.90, 1.00 | 0.075 | 0.53 | 0.39, 0.72 | <0.001 |
| **Household income per consumption unit** |  |  |  |  |  |  |
| < 980 €/month (Ref.) | — | — |  | — | — |  |
| 980 to 1,722€/month | 0.98 | 0.94, 1.02 | 0.3 | 1.16 | 0.91, 1.49 | 0.2 |
| 1,723 to 2,550 €/month | 0.99 | 0.95, 1.03 | 0.6 | 1.17 | 0.86, 1.60 | 0.3 |
| > 2,550 €/month | 1.01 | 0.96, 1.05 | 0.8 | 1.66 | 1.16, 2.39 | 0.006 |
| Refuse to answer | 1.01 | 0.96, 1.06 | 0.7 | 1.08 | 0.74, 1.59 | 0.7 |
| **Home location** |  |  |  |  |  |  |
| Montpellier city-center (Ref.) | — | — |  | — | — |  |
| Pericentral neighborhoods of Montpellier | 0.89 | 0.86, 0.93 | <0.001 | 1.11 | 0.87, 1.41 | 0.4 |
| Peri-urban area | 0.81 | 0.77, 0.85 | <0.001 | 1.69 | 1.29, 2.21 | <0.001 |
| **Car ownership** |  |  |  |  |  |  |
| No (Ref.) | — | — |  | — | — |  |
| Yes | 0.95 | 0.91, 0.98 | 0.006 | 1.60 | 1.31, 1.97 | <0.001 |

*^1^ CI = Confidence Interval^2^ Listed as ‘employee’, and ‘self-employed’*

*^3^ Listed as ‘jobless’, ‘homemaker’, ‘retired’, and ‘sick leave’*

Table 3: Variation in number of food stores according to households’ characteristics: comparison between around home and activity space measures

|  | **Around home (**Nagelkerke’s R² = 0.408) | | | | | | | **Activity Space (**Nagelkerke’s R² = 0.238) | | | | | |
| --- | --- | --- | --- | --- | --- | --- | --- | --- | --- | --- | --- | --- | --- |
|  | **Tercile 2** | | | **Tercile 3** | | | | **Tercile 2** | | | **Tercile 3** | | |
|  | OR^1^ | 95% CI^1^ | p-value | OR^1^ | 95% CI^1^ | p-value | OR^1^ | | 95% CI^1^ | p-value | OR^1^ | 95% CI^1^ | p-value |
| **Age of household head** |  |  |  |  |  |  |  | |  |  |  |  |  |
| < 35 years (Ref.) | — | — |  | — | — |  | — | | — |  | — | — |  |
| 35 to 50 years | 0.82 | 0.43, 1.55 | 0.5 | 0.53 | 0.25, 1.12 | 0.10 | 1.20 | | 0.63, 2.29 | 0.6 | 1.04 | 0.54, 1.98 | >0.9 |
| > 50 years | 0.46 | 0.25, 0.85 | 0.014 | 0.54 | 0.27, 1.09 | 0.085 | 1.50 | | 0.82, 2.75 | 0.2 | 1.07 | 0.58, 1.97 | 0.8 |
| **Education level of household head** |  |  |  |  |  |  |  | |  |  |  |  |  |
| High school diploma or lower (Ref.) | — | — |  | — | — |  | — | | — |  | — | — |  |
| Undergraduate degree | 0.71 | 0.42, 1.21 | 0.2 | 0.62 | 0.33, 1.16 | 0.13 | 0.99 | | 0.59, 1.66 | >0.9 | 2.01 | 1.11, 3.61 | 0.020 |
| Postgraduate degree | 1.08 | 0.61, 1.89 | 0.8 | 0.98 | 0.50, 1.92 | >0.9 | 1.29 | | 0.74, 2.24 | 0.4 | 2.66 | 1.43, 4.96 | 0.002 |
| **Household composition** |  |  |  |  |  |  |  | |  |  |  |  |  |
| One adult (Ref.) | — | — |  | — | — |  | — | | — |  | — | — |  |
| Multiple adults with at least one child | 0.82 | 0.47, 1.45 | 0.5 | 1.52 | 0.75, 3.07 | 0.2 | 1.20 | | 0.66, 2.20 | 0.5 | 1.49 | 0.80, 2.75 | 0.2 |
| Multiple adults | 0.81 | 0.49, 1.33 | 0.4 | 0.84 | 0.46, 1.54 | 0.6 | 0.79 | | 0.47, 1.30 | 0.4 | 0.89 | 0.52, 1.50 | 0.7 |
| One adult with at least one child | 1.38 | 0.62, 3.07 | 0.4 | 3.29 | 1.34, 8.04 | 0.009 | 1.57 | | 0.73, 3.38 | 0.2 | 1.74 | 0.79, 3.84 | 0.2 |
| **Employment status of household head** |  |  |  |  |  |  |  | |  |  |  |  |  |
| Employed^2^ (Ref.) | — | — |  | — | — |  | — | | — |  | — | — |  |
| Unemployed^3^ | 1.13 | 0.64, 2.00 | 0.7 | 1.88 | 0.98, 3.62 | 0.059 | 0.34 | | 0.20, 0.58 | <0.001 | 0.24 | 0.13, 0.43 | <0.001 |
| Student | 1.02 | 0.39, 2.70 | >0.9 | 0.24 | 0.08, 0.75 | 0.014 | 0.19 | | 0.07, 0.50 | <0.001 | 0.11 | 0.04, 0.30 | <0.001 |
| **Household income per consumption unit** |  |  |  |  |  |  |  | |  |  |  |  |  |
| < 980 €/month (Ref.) | — | — |  | — | — |  | — | | — |  | — | — |  |
| 980 to 1,722€/month | 1.01 | 0.53, 1.95 | >0.9 | 1.14 | 0.55, 2.36 | 0.7 | 1.72 | | 0.92, 3.20 | 0.089 | 0.79 | 0.40, 1.54 | 0.5 |
| 1,723 to 2,550 €/month | 0.54 | 0.27, 1.09 | 0.085 | 0.72 | 0.32, 1.60 | 0.4 | 0.84 | | 0.42, 1.69 | 0.6 | 0.58 | 0.29, 1.18 | 0.13 |
| > 2,550 €/month | 0.88 | 0.44, 1.77 | 0.7 | 0.56 | 0.24, 1.32 | 0.2 | 1.31 | | 0.63, 2.72 | 0.5 | 1.32 | 0.63, 2.74 | 0.5 |
| Refuse to answer | 0.80 | 0.33, 1.94 | 0.6 | 1.13 | 0.42, 3.05 | 0.8 | 1.29 | | 0.56, 3.00 | 0.6 | 0.99 | 0.40, 2.47 | >0.9 |
| **Home location** |  |  |  |  |  |  |  | |  |  |  |  |  |
| Montpellier city-center (Ref.) | — | — |  | — | — |  | — | | — |  | — | — |  |
| Pericentral neighborhoods of Montpellier | 0.79 | 0.32, 1.95 | 0.6 | 0.09 | 0.04, 0.20 | <0.001 | 0.30 | | 0.15, 0.60 | <0.001 | 0.22 | 0.11, 0.43 | <0.001 |
| Peri-urban area | 0.32 | 0.13, 0.80 | 0.015 | 0.02 | 0.01, 0.04 | <0.001 | 0.22 | | 0.11, 0.45 | <0.001 | 0.12 | 0.06, 0.25 | <0.001 |
| **Car ownership** |  |  |  |  |  |  |  | |  |  |  |  |  |
| No (Ref.) | — | — |  | — | — |  | — | | — |  | — | — |  |
| Yes | 0.87 | 0.42, 1.82 | 0.7 | 0.30 | 0.14, 0.64 | 0.002 | 1.03 | | 0.56, 1.89 | >0.9 | 1.01 | 0.53, 1.92 | >0.9 |

*^1^ OR = Odds Ratio, CI = Confidence Interval*

*^2^ Listed as ‘employee’, and ‘self-employed’*

*^3^ Listed as ‘jobless’, ‘homemaker’, ‘retired’, and ‘sick leave’*

Table 4: Variation in number of restaurants according to households’ characteristics: comparison between around home and activity space measures

|  | **Around home (**Nagelkerke’s R² = 0.372) | | | | | | | **Activity Space (**Nagelkerke’s R² = 0.246) | | | | | |
| --- | --- | --- | --- | --- | --- | --- | --- | --- | --- | --- | --- | --- | --- |
|  | **Tercile 2** | | | **Tercile 3** | | | | **Tercile 2** | | | **Tercile 3** | | |
|  | OR^1^ | 95% CI^1^ | p-value | OR^1^ | 95% CI^1^ | p-value | OR^1^ | | 95% CI^1^ | p-value | OR^1^ | 95% CI^1^ | p-value |
| **Age of household head** |  |  |  |  |  |  |  | |  |  |  |  |  |
| < 35 years (Ref.) | — | — |  | — | — |  | — | | — |  | — | — |  |
| 35 to 50 years | 0.53 | 0.27, 1.02 | 0.056 | 0.56 | 0.28, 1.10 | 0.092 | 1.30 | | 0.68, 2.50 | 0.4 | 0.98 | 0.50, 1.92 | >0.9 |
| > 50 years | 0.42 | 0.22, 0.78 | 0.006 | 0.37 | 0.19, 0.70 | 0.002 | 0.94 | | 0.51, 1.71 | 0.8 | 0.66 | 0.35, 1.24 | 0.2 |
| **Education level of household head** |  |  |  |  |  |  |  | |  |  |  |  |  |
| High school diploma or lower (Ref.) | — | — |  | — | — |  | — | | — |  | — | — |  |
| Undergraduate degree | 0.58 | 0.34, 1.01 | 0.052 | 0.87 | 0.48, 1.56 | 0.6 | 0.70 | | 0.42, 1.18 | 0.2 | 1.55 | 0.86, 2.81 | 0.15 |
| Postgraduate degree | 0.54 | 0.30, 0.96 | 0.036 | 0.73 | 0.39, 1.38 | 0.3 | 0.78 | | 0.45, 1.35 | 0.4 | 1.73 | 0.92, 3.27 | 0.088 |
| **Household composition** |  |  |  |  |  |  |  | |  |  |  |  |  |
| One adult (Ref.) | — | — |  | — | — |  | — | | — |  | — | — |  |
| Multiple adults with at least one child | 1.04 | 0.58, 1.85 | >0.9 | 0.73 | 0.38, 1.41 | 0.3 | 0.75 | | 0.41, 1.36 | 0.3 | 1.12 | 0.61, 2.07 | 0.7 |
| Multiple adults | 0.53 | 0.31, 0.92 | 0.023 | 0.67 | 0.39, 1.15 | 0.14 | 0.81 | | 0.49, 1.35 | 0.4 | 0.94 | 0.55, 1.60 | 0.8 |
| One adult with at least one child | 1.36 | 0.62, 2.97 | 0.4 | 1.54 | 0.69, 3.45 | 0.3 | 2.67 | | 1.25, 5.67 | 0.011 | 1.06 | 0.43, 2.58 | >0.9 |
| **Employment status of household head** |  |  |  |  |  |  |  | |  |  |  |  |  |
| Employed^2^ (Ref.) | — | — |  | — | — |  | — | | — |  | — | — |  |
| Unemployed^3^ | 0.75 | 0.41, 1.37 | 0.3 | 1.24 | 0.67, 2.28 | 0.5 | 0.36 | | 0.21, 0.61 | <0.001 | 0.31 | 0.17, 0.57 | <0.001 |
| Student | 0.92 | 0.34, 2.48 | 0.9 | 0.40 | 0.14, 1.13 | 0.084 | 0.23 | | 0.09, 0.58 | 0.002 | 0.13 | 0.05, 0.35 | <0.001 |
| **Household income per consumption unit** |  |  |  |  |  |  |  | |  |  |  |  |  |
| < 980 €/month (Ref.) | — | — |  | — | — |  | — | | — |  | — | — |  |
| 980 to 1,722€/month | 0.60 | 0.31, 1.15 | 0.12 | 0.81 | 0.42, 1.59 | 0.5 | 1.86 | | 1.00, 3.48 | 0.051 | 1.04 | 0.53, 2.04 | >0.9 |
| 1,723 to 2,550 €/month | 0.78 | 0.38, 1.58 | 0.5 | 0.93 | 0.44, 1.95 | 0.8 | 1.37 | | 0.69, 2.71 | 0.4 | 0.75 | 0.37, 1.55 | 0.4 |
| > 2,550 €/month | 0.74 | 0.36, 1.51 | 0.4 | 0.82 | 0.38, 1.77 | 0.6 | 1.80 | | 0.87, 3.71 | 0.11 | 1.72 | 0.82, 3.59 | 0.2 |
| Refuse to answer | 0.81 | 0.33, 2.01 | 0.7 | 1.16 | 0.46, 2.93 | 0.8 | 2.60 | | 1.14, 5.94 | 0.023 | 1.30 | 0.50, 3.33 | 0.6 |
| **Home location** |  |  |  |  |  |  |  | |  |  |  |  |  |
| Montpellier city-center (Ref.) | — | — |  | — | — |  | — | | — |  | — | — |  |
| Pericentral neighborhoods of Montpellier | 1.39 | 0.61, 3.18 | 0.4 | 0.16 | 0.08, 0.30 | <0.001 | 0.29 | | 0.14, 0.60 | <0.001 | 0.14 | 0.07, 0.28 | <0.001 |
| Peri-urban area | 0.43 | 0.18, 0.99 | 0.048 | 0.04 | 0.02, 0.08 | <0.001 | 0.21 | | 0.10, 0.46 | <0.001 | 0.08 | 0.04, 0.18 | <0.001 |
| **Car ownership** |  |  |  |  |  |  |  | |  |  |  |  |  |
| No (Ref.) | — | — |  | — | — |  | — | | — |  | — | — |  |
| Yes | 1.12 | 0.55, 2.31 | 0.8 | 0.43 | 0.22, 0.84 | 0.013 | 0.88 | | 0.48, 1.64 | 0.7 | 0.84 | 0.43, 1.62 | 0.6 |

*^1^ OR = Odds Ratio, CI = Confidence Interval*

*^2^ Listed as ‘employee’, and ‘self-employed’*

*^3^ Listed as ‘jobless’, ‘homemaker’, ‘retired’, and ‘sick leave’’*

Table 5: Variation in diversity of food stores according to households’ characteristics: comparison between around home and activity space measures

|  | **Around home (**Nagelkerke’s R² = 0.338) | | | | | | | **Activity Space (**Nagelkerke’s R² = 0.243) | | | | | |
| --- | --- | --- | --- | --- | --- | --- | --- | --- | --- | --- | --- | --- | --- |
|  | **3 to 5 food stores** | | | **6 to 8 food stores** | | | | **3 to 5 food stores** | | | **6 to 8 food stores** | | |
|  | OR^1^ | 95% CI^1^ | p-value | OR^1^ | 95% CI^1^ | p-value | OR^1^ | | 95% CI^1^ | p-value | OR^1^ | 95% CI^1^ | p-value |
| **Age of household head** |  |  |  |  |  |  |  | |  |  |  |  |  |
| < 35 years (Ref.) | — | — |  | — | — |  | — | | — |  | — | — |  |
| 35 to 50 years | 1.16 | 0.64, 2.10 | 0.6 | 0.37 | 0.17, 0.82 | 0.015 | 0.79 | | 0.24, 2.61 | 0.7 | 0.71 | 0.23, 2.19 | 0.6 |
| > 50 years | 0.62 | 0.35, 1.10 | 0.10 | 0.29 | 0.14, 0.61 | 0.001 | 0.97 | | 0.32, 2.98 | >0.9 | 0.97 | 0.34, 2.79 | >0.9 |
| **Education level of household head** |  |  |  |  |  |  |  | |  |  |  |  |  |
| High school diploma or lower (Ref.) | — | — |  | — | — |  | — | | — |  | — | — |  |
| Undergraduate degree | 0.84 | 0.51, 1.38 | 0.5 | 1.00 | 0.48, 2.09 | >0.9 | 1.08 | | 0.50, 2.30 | 0.9 | 1.60 | 0.76, 3.38 | 0.2 |
| Postgraduate degree | 1.13 | 0.67, 1.91 | 0.7 | 1.05 | 0.46, 2.36 | >0.9 | 0.48 | | 0.21, 1.11 | 0.085 | 1.15 | 0.53, 2.51 | 0.7 |
| **Household composition** |  |  |  |  |  |  |  | |  |  |  |  |  |
| One adult (Ref.) | — | — |  | — | — |  | — | | — |  | — | — |  |
| Multiple adults with at least one child | 0.77 | 0.44, 1.33 | 0.3 | 1.32 | 0.59, 2.97 | 0.5 | 0.85 | | 0.29, 2.48 | 0.8 | 1.04 | 0.38, 2.81 | >0.9 |
| Multiple adults | 0.80 | 0.50, 1.29 | 0.4 | 1.02 | 0.51, 2.02 | >0.9 | 0.56 | | 0.26, 1.18 | 0.13 | 0.49 | 0.24, 0.98 | 0.045 |
| One adult with at least one child | 1.96 | 1.02, 3.78 | 0.045 | 0.84 | 0.29, 2.47 | 0.8 | 4.81 | | 0.73, 31.7 | 0.10 | 4.11 | 0.63, 26.8 | 0.14 |
| **Employment status of household head** |  |  |  |  |  |  |  | |  |  |  |  |  |
| Employed^2^ (Ref.) | — | — |  | — | — |  | — | | — |  | — | — |  |
| Unemployed^3^ | 1.66 | 0.98, 2.81 | 0.058 | 2.35 | 1.08, 5.09 | 0.031 | 0.28 | | 0.12, 0.64 | 0.003 | 0.16 | 0.07, 0.34 | <0.001 |
| Student | 0.54 | 0.23, 1.26 | 0.2 | 0.17 | 0.05, 0.54 | 0.003 | 0.47 | | 0.12, 1.87 | 0.3 | 0.08 | 0.02, 0.30 | <0.001 |
| **Household income per consumption unit** |  |  |  |  |  |  |  | |  |  |  |  |  |
| < 980 €/month (Ref.) | — | — |  | — | — |  | — | | — |  | — | — |  |
| 980 to 1,722€/month | 0.84 | 0.47, 1.51 | 0.6 | 0.73 | 0.33, 1.57 | 0.4 | 1.76 | | 0.64, 4.81 | 0.3 | 1.39 | 0.52, 3.66 | 0.5 |
| 1,723 to 2,550 €/month | 0.67 | 0.35, 1.29 | 0.2 | 0.64 | 0.27, 1.51 | 0.3 | 0.98 | | 0.33, 2.86 | >0.9 | 0.64 | 0.23, 1.78 | 0.4 |
| > 2,550 €/month | 0.88 | 0.46, 1.69 | 0.7 | 0.32 | 0.12, 0.88 | 0.027 | 0.81 | | 0.26, 2.52 | 0.7 | 1.05 | 0.37, 2.99 | >0.9 |
| Refuse to answer | 0.97 | 0.44, 2.13 | >0.9 | 0.43 | 0.13, 1.45 | 0.2 | 0.91 | | 0.30, 2.79 | 0.9 | 0.60 | 0.20, 1.79 | 0.4 |
| **Home location** |  |  |  |  |  |  |  | |  |  |  |  |  |
| Montpellier city-center (Ref.) | — | — |  | — | — |  | — | | — |  | — | — |  |
| Pericentral neighborhoods of Montpellier | 0.45 | 0.24, 0.82 | 0.010 | 0.06 | 0.03, 0.12 | <0.001 | 0.15 | | 0.03, 0.84 | 0.031 | 0.10 | 0.02, 0.53 | 0.007 |
| Peri-urban area | 0.21 | 0.11, 0.39 | <0.001 | 0.03 | 0.02, 0.07 | <0.001 | 0.05 | | 0.01, 0.31 | 0.001 | 0.04 | 0.01, 0.25 | <0.001 |
| **Car ownership** |  |  |  |  |  |  |  | |  |  |  |  |  |
| No (Ref.) | — | — |  | — | — |  | — | | — |  | — | — |  |
| Yes | 0.43 | 0.24, 0.77 | 0.005 | 0.48 | 0.23, 1.01 | 0.054 | 2.14 | | 0.88, 5.24 | 0.095 | 2.42 | 1.00, 5.83 | 0.049 |

*^1^ OR = Odds Ratio, CI = Confidence Interval*

*^2^ Listed as ‘employee’, and ‘self-employed’*

*^3^ Listed as ‘jobless’, ‘homemaker’, ‘retired’, and ‘sick leave’*

Table 6: Variation in relative density of F&V stores according to households’ characteristics: comparison between around home and activity space measures

|  | **Around home** | | | **Activity space** | | |
| --- | --- | --- | --- | --- | --- | --- |
|  | OR^1^ | 95% CI^1^ | p-value | OR^1^ | 95% CI^1^ | p-value |
| **Age of household head** |  |  |  |  |  |  |
| < 35 years (Ref.) | — | — |  | — | — |  |
| 35 to 50 years | 1.07 | 0.58, 2.00 | 0.8 | 1.24 | 0.61, 2.52 | 0.6 |
| > 50 years | 0.59 | 0.32, 1.08 | 0.087 | 1.01 | 0.51, 1.99 | >0.9 |
| **Education level of household head** |  |  |  |  |  |  |
| High school diploma or lower (Ref.) | — | — |  | — | — |  |
| Undergraduate degree | 0.53 | 0.32, 0.87 | 0.013 | 0.55 | 0.33, 0.94 | 0.028 |
| Postgraduate degree | 0.97 | 0.57, 1.64 | >0.9 | 1.18 | 0.66, 2.11 | 0.6 |
| **Household composition** |  |  |  |  |  |  |
| One adult (Ref.) | — | — |  | — | — |  |
| Multiple adults with at least one child | 0.70 | 0.37, 1.31 | 0.3 | 0.66 | 0.33, 1.32 | 0.2 |
| Multiple adults | 1.19 | 0.73, 1.92 | 0.5 | 0.82 | 0.48, 1.39 | 0.5 |
| One adult with at least one child | 0.58 | 0.22, 1.48 | 0.3 | 0.39 | 0.14, 1.09 | 0.072 |
| **Employment status of household head** |  |  |  |  |  |  |
| Employed^2^ (Ref.) | — | — |  | — | — |  |
| Unemployed^3^ | 0.90 | 0.48, 1.68 | 0.7 | 0.35 | 0.19, 0.63 | <0.001 |
| Student | 0.77 | 0.35, 1.69 | 0.5 | 0.41 | 0.17, 0.98 | 0.044 |
| **Household income per consumption unit** |  |  |  |  |  |  |
| < 980 €/month (Ref.) | — | — |  | — | — |  |
| 980 to 1,722€/month | 1.21 | 0.70, 2.10 | 0.5 | 1.21 | 0.65, 2.24 | 0.6 |
| 1,723 to 2,550 €/month | 0.84 | 0.46, 1.55 | 0.6 | 0.72 | 0.36, 1.42 | 0.3 |
| > 2,550 €/month | 0.66 | 0.33, 1.32 | 0.2 | 0.58 | 0.28, 1.23 | 0.2 |
| Refuse to answer | 0.99 | 0.48, 2.02 | >0.9 | 1.04 | 0.46, 2.38 | >0.9 |
| **Home location** |  |  |  |  |  |  |
| Montpellier city-center (Ref.) | — | — |  | — | — |  |
| Pericentral neighborhoods of Montpellier | 0.14 | 0.07, 0.27 | <0.001 | 0.13 | 0.05, 0.32 | <0.001 |
| Peri-urban area | 0.09 | 0.04, 0.18 | <0.001 | 0.14 | 0.05, 0.39 | <0.001 |
| **Car ownership** |  |  |  |  |  |  |
| No (Ref.) | — | — |  | — | — |  |
| Yes | 1.03 | 0.59, 1.80 | >0.9 | 1.44 | 0.76, 2.73 | 0.3 |

*^1^ OR = Odds Ratio, CI = Confidence Interval*

*^2^ Listed as ‘employee’, and ‘self-employed’*

*^3^ Listed as ‘jobless’, ‘homemaker’, ‘retired’, and ‘sick leave’*

Table 7: Variation in relative density of fast food restaurants according to households’ characteristics: comparison between around home and activity space measures

|  | **Around home** | | | **Activity space** | | |
| --- | --- | --- | --- | --- | --- | --- |
|  | OR^1^ | 95% CI^1^ | p-value | OR^1^ | 95% CI^1^ | p-value |
| **Age of household head** |  |  |  |  |  |  |
| < 35 years (Ref.) | — | — |  | — | — |  |
| 35 to 50 years | 0.95 | 0.56, 1.61 | 0.8 | 1.01 | 0.62, 1.63 | >0.9 |
| > 50 years | 0.97 | 0.54, 1.73 | >0.9 | 2.16 | 1.27, 3.68 | 0.005 |
| **Education level of household head** |  |  |  |  |  |  |
| High school diploma or lower (Ref.) | — | — |  | — | — |  |
| Undergraduate degree | 0.90 | 0.53, 1.53 | 0.7 | 0.60 | 0.36, 1.00 | 0.049 |
| Postgraduate degree | 0.65 | 0.36, 1.15 | 0.14 | 0.65 | 0.37, 1.16 | 0.14 |
| **Employment status of household head** |  |  |  |  |  |  |
| Employed^2^ (Ref.) | — | — |  | — | — |  |
| Unemployed^3^ | 0.98 | 0.55, 1.73 | >0.9 | 0.42 | 0.25, 0.73 | 0.002 |
| Student | 1.41 | 0.60, 3.28 | 0.4 | 1.34 | 0.64, 2.81 | 0.4 |
| **Household income per consumption unit** |  |  |  |  |  |  |
| < 980 €/month (Ref.) | — | — |  | — | — |  |
| 980 to 1,722€/month | 0.80 | 0.46, 1.38 | 0.4 | 0.98 | 0.58, 1.66 | >0.9 |
| 1,723 to 2,550 €/month | 0.64 | 0.35, 1.15 | 0.14 | 0.79 | 0.44, 1.41 | 0.4 |
| > 2,550 €/month | 1.00 | 0.53, 1.89 | >0.9 | 0.69 | 0.37, 1.30 | 0.3 |
| Refuse to answer | 0.52 | 0.23, 1.14 | 0.10 | 0.57 | 0.27, 1.22 | 0.15 |
| **Home location** |  |  |  |  |  |  |
| Montpellier city-center (Ref.) | — | — |  | — | — |  |
| Pericentral neighborhoods of Montpellier | 3.25 | 1.90, 5.55 | <0.001 | 2.59 | 1.56, 4.30 | <0.001 |
| Peri-urban area | 0.82 | 0.49, 1.37 | 0.4 | 1.35 | 0.81, 2.25 | 0.2 |

*^1^ OR = Odds Ratio, CI = Confidence Interval*

*^2^ Listed as ‘employee’, and ‘self-employed’*

*^3^ Listed as ‘jobless’, ‘homemaker’, ‘retired’, and ‘sick leave’*

Table 7: Correlation measures of the food environment between around home and activity spaces

|  | Size of area of exposure | | Number of food outlets | | Number of restaurants | | Diversity of food stores | | Relative density of F&V stores | | Relative density of fast food restaurants | |
| --- | --- | --- | --- | --- | --- | --- | --- | --- | --- | --- | --- | --- |
|  | Rho | p-value | Rho | p-value | Rho | p-value | Rho | p-value | Rho | p-value | Rho | p-value |
| **Age of household head** |  |  |  |  |  |  |  |  |  |  |  |  |
| < 35 years (Ref.) | -0.104 | 0.107 | 0.348 | <0.001 | 0.451 | <0.001 | 0.312 | <0.001 | 0.573 | <0.001 | 0.595 | <0.001 |
| 35 to 50 years | -0.177 | 0.010 | 0.187 | 0.02 | 0.243 | 0.02 | 0.155 | 0.02 | 0.548 | <0.001 | 0.391 | <0.001 |
| > 50 years | -0.115 | 0.081 | 0.281 | <0.001 | 0.265 | <0.001 | 0.220 | <0.001 | 0.584 | <0.001 | 0.436 | <0.001 |
| **Education level of household head** |  |  |  |  |  |  |  |  |  |  |  |  |
| High school diploma or lower (Ref.) | -0.122 | 0.112 | 0.268 | 0.001 | 0.207 | <0.001 | 0.247 | 0.001 | 0.682 | <0.001 | 0.522 | <0.001 |
| Undergraduate degree | -0.126 | 0.043 | 0.269 | <0.001 | 0.352 | <0.001 | 0.215 | <0.001 | 0.475 | <0.001 | 0.538 | <0.001 |
| Postgraduate degree | -0.215 | <0.001 | 0.273 | <0.001 | 0.362 | <0.001 | 0.200 | <0.001 | 0.555 | <0.001 | 0.316 | <0.001 |
| **Household composition** |  |  |  |  |  |  |  |  |  |  |  |  |
| One adult (Ref.) | -0.109 | 0.098 | 0.414 | <0.001 | 0.421 | <0.001 | 0.340 | <0.001 | 0.541 | <0.001 | 0.518 | <0.001 |
| Multiple adults with at least one child | -0.205 | 0.008 | 0.138 | 0.25 | 0.203 | 0.25 | 0.090 | 0.25 | 0.633 | <0.001 | 0.434 | <0.001 |
| Multiple adults | -0.091 | 0.143 | 0.298 | <0.001 | 0.378 | <0.001 | 0.249 | <0.001 | 0.520 | <0.001 | 0.480 | <0.001 |
| One adult with at least one child | -0.242 | 0.161 | 0.260 | 0.07 | 0.391 | 0.07 | 0.313 | 0.07 | 0.658 | <0.001 | 0.391 | <0.001 |
| **Employment status of household head** |  |  |  |  |  |  |  |  |  |  |  |  |
| Employed^2^ (Ref.) | -0.244 | <0.001 | 0.223 | <0.001 | 0.299 | <0.001 | 0.167 | <0.001 | 0.547 | <0.001 | 0.380 | <0.001 |
| Unemployed^3^ | 0.138 | 0.114 | 0.512 | <0.001 | 0.564 | <0.001 | 0.454 | <0.001 | 0.663 | <0.001 | 0.614 | <0.001 |
| Student | 0.285 | 0.014 | 0.635 | <0.001 | 0.595 | <0.001 | 0.616 | <0.001 | 0.697 | <0.001 | 0.701 | <0.001 |
| **Household income per consumption unit** |  |  |  |  |  |  |  |  |  |  |  |  |
| < 980 €/month (Ref.) | -0.003 | 0.968 | 0.413 | <0.001 | 0.449 | <0.001 | 0.390 | <0.001 | 0.665 | <0.001 | 0.634 | <0.001 |
| 980 to 1,722€/month | -0.239 | 0.001 | 0.143 | 0.12 | 0.288 | 0.12 | 0.117 | 0.12 | 0.535 | <0.001 | 0.349 | <0.001 |
| 1,723 to 2,550 €/month | -0.141 | 0.098 | 0.394 | 0.001 | 0.332 | 0.001 | 0.269 | 0.001 | 0.428 | <0.001 | 0.526 | <0.001 |
| > 2,550 €/month | -0.003 | 0.978 | 0.283 | 0.03 | 0.318 | 0.03 | 0.200 | 0.03 | 0.574 | <0.001 | 0.306 | <0.001 |
| Refuse to answer | -0.155 | 0.289 | 0.276 | 0.02 | 0.381 | 0.02 | 0.332 | 0.02 | 0.656 | <0.001 | 0.513 | <0.001 |
| **Home location** |  |  |  |  |  |  |  |  |  |  |  |  |
| Montpellier city-center (Ref.) | -0.0283 | 0.749 | 0.523 | <0.001 | 0.603 | <0.001 | 0.497 | <0.001 | 0.617 | <0.001 | 0.639 | <0.001 |
| Pericentral neighborhoods of Montpellier | -0.050 | 0.404 | 0.125 | 0.02 | 0.141 | 0.02 | 0.134 | 0.02 | 0.527 | <0.001 | 0.437 | <0.001 |
| Peri-urban area | -0.037 | 0.539 | 0.157 | 0.002 | 0.186 | 0.002 | 0.187 | 0.002 | 0.517 | <0.001 | 0.260 | <0.001 |
| **Car ownership** |  |  |  |  |  |  |  |  |  |  |  |  |
| No (Ref.) | 0.228 | 0.008 | 0.683 | <0.001 | 0.702 | <0.001 | 0.647 | <0.001 | 0.716 | <0.001 | 0.699 | <0.001 |
| Yes | -0.116 | 0.006 | 0.233 | <0.001 | 0.275 | <0.001 | 0.197 | <0.001 | 0.532 | <0.001 | 0.402 | <0.001 |
